# New Predictor of Organ Failure in Acute Pancreatitis: CD4+ T Lymphocytes and CD19+ B Lymphocytes

**DOI:** 10.1155/2018/1012584

**Published:** 2018-12-05

**Authors:** Chenyuan Shi, Chaoqun Hou, Xiaole Zhu, Yunpeng Peng, Feng Guo, Kai Zhang, Dongya Huang, Qiang Li, Yi Miao

**Affiliations:** ^1^Pancreas Centre, First Affiliated Hospital of Nanjing Medical University, Nanjing, Jiangsu, Province, China; ^2^Pancreas Institute, Nanjing Medical University, Nanjing, Jiangsu Province, China

## Abstract

**Objective:**

Lymphocytes are one of the main effector cells in the inflammatory response of acute pancreatitis (AP). The purpose of the study was to evaluate whether peripheral blood lymphocyte (PBL) subsets at admission change during AP based on clinical outcomes and to explore whether these changes vary by aetiology of AP. Hence, we performed a prospective study to find a predictor in lymphocyte subsets that might allow easier, earlier, and more accurate prediction of clinical outcomes.

**Methods:**

Patients with AP were enrolled from December 2017 to June 2018 at the First Affiliated Hospital of Nanjing Medical University. Age, sex, clinical and biochemical parameters, and aetiology of AP were obtained at admission. PBL counts were assessed within 24 hours after admission. Clinical outcomes were observed as endpoints. The areas under the curve (AUCs) of different predictors were calculated using the receiver operating characteristic (ROC) curve.

**Results:**

Overall, 133 patients were included. Patients (n=24) with organ failure (OF) had significantly lower CD4+ T lymphocyte levels than those (n=109) with No OF (NOF) (39.60 (33.94-46.13) vs. 32.41 (26.51-38.00), P=0.004). The OF group exhibited significantly higher CD19+ B lymphocytes than the NOF group (16.07 (10.67-21.06) vs. 23.78 (17.84-29.45), P=0.001). Of the AP cases, 68.8% were caused by gallstones; 10.1% were attributed to alcohol; 16.5% were due to hyperlipidaemia; and 4.6% had other causes. Across all aetiologies, a lower CD4+ T lymphocyte level was significantly related to OF (P<0.05). However, CD19+ B lymphocytes were significant only in gallstone pancreatitis (P<0.05). The ROC curve results showed that the AUC values of CD4+T lymphocytes, CD19+ B lymphocytes, and combined CD4+T lymphocytes and CD19+ B lymphocytes were similar to those of traditional scoring systems, such as APACHEII and Ranson.

**Conclusions:**

CD4+ T and CD19+ B lymphocytes during the early phase of AP can predict OF.

## 1. Introduction

Acute pancreatitis (AP) is one of the most common diseases of the digestive system. Outside of China, the cause of AP is mostly due to excessive alcohol intake, while in China, many cases are caused by gallbladder or biliary stones [1]. Currently, with improvements of living standards, pancreatitis caused by hyperlipidaemia has also shown a clear upward trend. According to the 2012 Revised Atlanta classification, AP is divided into mild (MAP), moderately severe (MSAP), and severe (SAP) categories [2]. MAP is not often associated with organ failure (OF), so the mortality is often less than 1%. Moderate or severe pancreatitis is often associated with transient or persistent organ failure, resulting in an increase in mortality of up to 10-30% [3]. Due to the large clinical differences in AP, multiple severity scoring systems have been used to assess AP patients, such as the acute physiological assessment and chronic health assessment II (APACHE II) score, acute pancreatitis severity bedside index (BISAP) score, Ranson score, and Glasgow-Imia criteria [4]. However, these scoring systems usually involve many variables that are not readily available. For example, the APACHE II score includes 12 clinical or biochemical parameters, so APACHE II scores are more detailed and the calculation is more complex; 11 variables need to be collected at admission and 48 hours after admission in the Ranson score. The Glasgow scoring system is derived from nine variables and requires 48 hours to complete [5, 6]. However, if the occurrence and development of OF in AP can be predicted early, early initiation and targeting of therapy can be undertaken as soon as possible to reduce complications. Prediction of the development of OF in patients can be performed with the modified Marshall scoring system [7].

The immune system has the role of immune surveillance, defence, and regulation. This system consists of immune organs, immune cells, and immunologically active substances and is divided into innate immunity (also known as nonspecific immunity) and adaptive immunity (also known as specific immunity), which is further divided into humoural immunity and cellular immunity [8]. Evidence suggests that there is an important relationship between the innate immune component of the pathogenesis of AP and the severity of the disease [9–11]. Neutrophils and macrophages serve as the first line of defence for the immune system, and T and B lymphocytes also play a central role in the immune response of the body. A large number of studies have reported the different inflammatory mediators that are produced in the early stage of AP and their effects on the body. However, the means by which the activation of lymphocyte subsets in the early stage of AP modulates the balance between proinflammatory and anti-inflammatory immune responses are still poorly understood. When immune function declines, the body is more prone to infectious complications and OF, although others have suggested that a reduction in CD4+T lymphocytes is valuable in a variety of inflammatory and immune diseases such as abdominal syndrome in AP patients [12]. However, these studies have some limitations; for example, the diagnosis of abdominal syndrome in AP was retrospective. Thus, we first observed whether peripheral blood lymphocyte subsets (i.e., CD3+Tlymphocytes, CD4+Tlymphocytes, CD8+cytotoxic T lymphocytes, CD16+CD56+ natural killer cells, CD19+Blymphocytes, and CD4+/CD8+ T lymphocytes) at admission changed in the early stage of AP in order to research the occurrence of AP. Second, we hypothesized that the activation of lymphocyte subsets is associated with different outcomes in AP patients in order to detect the development of AP. Therefore, we conducted this prospective observational survey.

## 2. Materials and Methods

### 2.1. Patients

We selected 133 AP patients who were admitted to the Pancreas Center at the First Affiliated Hospital of Nanjing Medical University from December 2017 to June 2018. We diagnosed AP according to the Revised Atlanta Classification 2012 as follows: (1) acute episodes of abdominal pain that often radiated to the back; (2) levels of serum amylase and lipase upwards of 3 times greater than normal levels; (3) the imaging examination was consistent with AP. Patients who presented at least two of these features were included.

Exclusion criteria included any of the following: (1) age less than 18 years old or more than 80 years old; (2) any surgery performed 3 days after admission; (3) previous or long-term use of immunosuppressive therapy; (4) innate impaired immune function; (5) history of tumour or chronic lung, kidney or cardiovascular disease; or (6) traumatic pancreatitis or chronic pancreatitis. The study was conducted in accordance with the principles of the Helsinki Declaration, and the study was approved by the First Affiliated Hospital of Nanjing Medical University.

### 2.2. Data Collection

All patients with AP were divided into four groups according to its aetiology. (1) gallstone pancreatitis, which is caused by gallstones or bile duct stones; (2) alcoholic pancreatitis, which is observed in patients with a history of drinking or recent alcohol intake; (3) hyperlipidaemia pancreatitis, in which the blood TG value exceeds 11.30 mmol/L or blood TG is between 5.65 and 11.30 mmol/L with white, opaqueserum; (4) other pancreatitis, which could not be diagnosed by medical history, physical examination, laboratory studies, or imaging methods.

Each patient's peripheral venous blood was collected within the first 24 hours after hospital admission. Peripheral blood lymphocyte subsets, including CD3+Tlymphocytes, CD4+Tlymphocytes, CD8+cytotoxicTlymphocytes, CD16+CD56+ natural killer cells, and CD19+ B lymphocytes of patients, were measured in the hospital laboratory. We recorded demographic data, clinical and biochemical parameters, and outcome.

### 2.3. Endpoint of Study and Definition of Organ Failure

The primary observational endpoint of the study was OF, which was evaluated by a modified Marshall scoring system. Patients who presented one or two of the following features were included: (1) definite renal failure, defined as serum creatinine of no more than 1.9 mg/dL; (2) cardiovascular failure, defined as a systolic blood pressure less than 90 mmHg, even after fluid replacement; and (3) respiratory failure, defined as a ratio of PaO2/FiO2 less than 300 mmHg [13].

### 2.4. Treatments

According to the British and Chinese Medical Association guidelines for the treatment of AP, all patients received standard treatment, including nutritional support, early fluid resuscitation, target organ treatment, and prophylactic antibiotics [14, 15].

### 2.5. Statistical Analysis

Statistical analysis was performed with SPSS 23.0. Continuous variables with a normal distribution are shown as the mean ± standard deviation (SD), and Student's t test was used to compare two groups. If variables were nonnormally distributed, data are presented as the median (25th-75th percentile), and a nonparametric Mann-Whitney U test was chosen. Categorical variables were compared using a chi-square test. P values less than 0.05 were considered to indicate significance. In addition, 95% confidence intervals (95% CIs) were obtained. A receiver operating characteristic (ROC) curve was constructed to predict organ failure, and the area under the curve (AUC) was used to analyse the ability of factors to predict OF.

## 3. Result

### 3.1. Peripheral Blood Lymphocyte Subsets of AP Patients

A total of 133 patients were included in this study. There were no patients lost to follow-up, and none of the patients had incomplete clinical data. The CD3+ T lymphocyte count was 66.00 (56.69-73.19), the CD4+T lymphocyte count was 38.20 (31.19-45.31), the CD8+cytotoxic T lymphocyte count was 21.62 (17.27-26.39), the CD16+CD56+ natural killer cell count was 12.07 (8.57-17.78), the CD19+B lymphocyte count was 16.80 (11.35-23.01), and the CD4+/CD8+ lymphocyte count was 1.79 (1.25-2.43). Except for CD19+B lymphocyte, the median of all peripheral blood lymphocyte subsets was in the normal range (Table 1).

### 3.2. Basic Characteristics and Peripheral Blood Lymphocyte Subsets in the OF and NOF Groups

Based on the 2012 Revised Atlanta classification, AP was divided into MAP and SAP. SAP was usually accompanied by OF. Therefore, patients were divided into two subgroups (OF group and NOF group) according to the clinical outcome of the presence or absence of OF. Baseline patient characteristics, including demographic data, clinical laboratory values at admission and different outcomes, are presented in Table 2. Twenty-four (18%) patients presented pulmonary and/or circulatory and/or renal complications. However, after appropriate treatment including multiple percutaneous, CT-guided external drainage procedures, no patient died in the hospital. The mean age was higher in the NOF group, but there were no significant differences in age. In 24 patients with OF, the biliary aetiology accounted for 66.7% (n=16); the alcoholic aetiology accounted for 8.3% (n=2); and the hyperlipidaemia aetiology accounted for 20.8% (n=5). Of the 109 NOF cases, 68.8% (n=75) were attributed to biliary aetiology, 10.1% (n=11) were ascribed to alcoholic aetiology, and 16.5% (n=18) were due to hyperlipidaemia aetiology. No difference in the aetiology of AP was found between the OF and NOF groups. Furthermore, no death was observed in either group.

The CD3+ T lymphocytes (66.50 (57.45-73.70) vs. 61.31 (51.18-72.38), P=0.133), CD8+cytotoxic Tlymphocytes (21.62 (17.27-26.18) vs. 21.63 (17.33-26.94), P=0.847), CD16+CD56+ natural killer cells (12.14 (8.93-17.96) vs. 11.07 (6.09-16.48), P=0.343), and CD4+/CD8+ (1.82 (1.30-2.51) vs. 1.61 (1.11-2.10), P=0.180) were similar between the NOF and OF groups. However, the CD4+Tlymphocyte count was significantly decreased in the OF group compared with that of the NOF group (39.60 (33.94-46.13) vs. 32.41 (26.51-38.00), P=0.004), and CD19+ B lymphocytes (16.07 (10.67-21.06) vs. 23.78 (17.84-29.45), P=0.001) were significantly higher in the OF group. (Table 3) The patients with OF typically spent more days in the hospital than did those with NOF. Therefore, we speculated that CD4+ T lymphocytes and CD19+ B lymphocytes can be used as predictors of OF.

### 3.3. Peripheral Blood Lymphocyte Subsets in Different Aetiologies

CD4+ lymphocytes and CD19+ lymphocytes were significantly different in all patients. We next investigated whether CD4+ T lymphocytes and CD19+ B lymphocytes were still significantly different in patients with AP with different pathogenesis. Gallstones, alcohol misuse, and hyperlipidaemia are the main risk factors for AP. Therefore, we performed a subgroup analysis (Tables 4, 5, and 6). CD3+CD4+T lymphocytes were significantly decreased across different aetiologies. However, a similar pattern was detected for CD19+B lymphocytes only in gallstone AP (Table 4), whereas this phenomenon was not significant in the alcoholic AP (Table 5) and hyperlipidaemia AP groups (Table 6).

### 3.4. Predictive Value of CD4+T Lymphocytes and CD19+ B Lymphocytes

**Figure 1:**
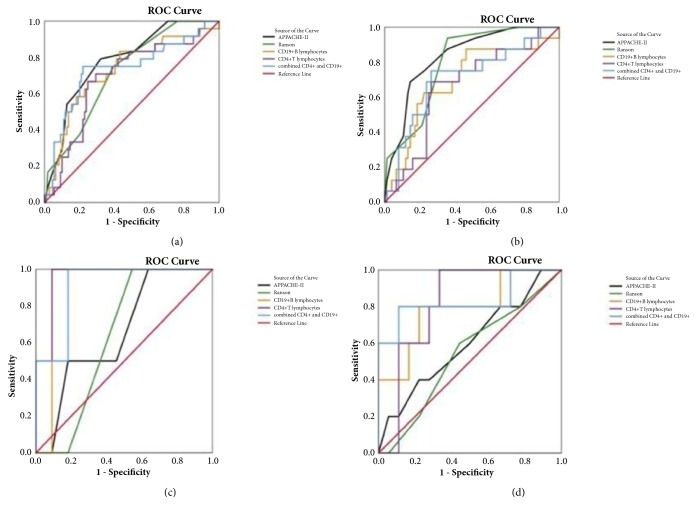
ROC curves to predict organ failure. (a) Total pancreatitis, (b) biliary pancreatitis, (c) alcoholic pancreatitis, and (d) hyperlipidemia pancreatitis.

The ROC was used to evaluate the diagnostic value of peripheral blood lymphocyte subsets for OF. For patients with OF, the AUCs of CD4+T lymphocytes and CD19+ B lymphocytes were calculated as follows (Figure 1): compared with a complex scoring system such as the Ranson score (AUROC 0.72) or APACHE II score (AUROC 0.78), CD4+T lymphocytes presented an AUC of 0.69, and CD19+ B lymphocytes showed an AUC of 0.72. To predict OF more accurately, the AUC was recalculated by combining CD4+T and CD19+ B lymphocytes as 0.73. To explore whether the predictive value still exists across different aetiologies of AP, the AUCs of different types of AP were calculated using the ROC curve as well. For biliary pancreatitis, CD4+T lymphocytes presented an AUC of 0.66, CD19+ B lymphocytes showed an AUC of 0.70, the combination of CD4+T and CD19+ B lymphocytes had an AUC of 0.71, and the AUC of APACHE II score and Ranson score were 0.83 and 0.80, respectively. In alcoholic pancreatitis, the CD4+T lymphocytes presented an AUC of 0.96, the CD19+ B lymphocytes showed an AUC of 0.91, the combination ofCD4+T and CD19+ B lymphocytes had an AUC of 0.91, and the AUC of the APACHE II score and Ranson score were 0.66 and 0.64, respectively. In hyperlipidaemia pancreatitis, the CD4+T lymphocytes presented an AUC of 0.81, the CD19+ B lymphocytes showed an AUC of 0.79, the combination of CD4+T and CD19+ B lymphocytes had an AUC of 0.83, and the AUC of the APACHE II score and Ranson score were 0.60 and 0.54, respectively (Table 7). In total, the ROC curve results showed that the AUC values of CD4+T lymphocytes, CD19+ B lymphocytes and the combination of CD4+T lymphocytes and CD19+ B lymphocytes had accuracies similar to those more complex scoring systems such as the Ranson and APACHE II scores.

## 4. Discussion

A few studies have investigated the participation of the innate immune system (macrophages, neutrophils, etc.) and other acquired immune systems (lymphocytes, etc.) in the immune response during the development of AP. Macrophages and neutrophils participate in AP's strong immune response by secreting a large number of inflammatory factors [16, 17]. Lymphocytes are white blood cells that are produced by lymphoid organs, and participate in the body's immune response function. Lymphocytes are a kind of cell line with immune recognition function. According to their function and surface molecules, they can be divided into T lymphocytes (T cells), B lymphocytes (B cells) and natural killer (NK) cells. T lymphocytes and B lymphocytes mediate cellular and humoural immunity, respectively. In recent years, a considerable amount of direct or indirect evidence has further confirmed that lymphocytes can not only promote the immune response but also eliminate pathogenic microorganisms. They also have an immune regulatory function that inhibits an excessive immune response[18]. Although a large number of studies exist on how substantial numbers of inflammatory factors are produced in the early stage of AP, there are still many gaps and controversies about how AP over-regulates the inflammatory response. From this perspective, we therefore designed our research. Our prospective study verifies, probably for the first time, an analysis of the relationship of peripheral blood lymphocyte subsets and OF with reference to the different aetiologies of AP. CD3+T lymphocytes, CD4+ Tlymphocytes, CD8+cytotoxicTlymphocytes, CD19+B lymphocytes and CD16+CD56+ NK cells were assessed on the first day after hospitalization, and clinical outcomes were followed. The principal findings were as follows: (1) Except for CD19+B lymphocyte, the median of the peripheral blood lymphocyte counts were all within the normal range at the occurrence of AP. (2) Accompanied by the development of AP, peripheral lymphocyte subsets of total AP patients and activated CD4+T and CD19+B lymphocytes were significantly correlated with OF. The lower the proportion of CD4+T lymphocytes and the higher the proportion of CD19+B lymphocytes at admission, the more likely OF is to occur in the later stage. Thus, these indicators can be used as a predictor of OF estimation in AP. (3) When considering different aetiologies of AP, there is also a statistically significant association between CD4+T lymphocytes and OF. However, CD19+B lymphocytes show a significant difference only in biliary pancreatitis. (4) The AUC value of CD4+T lymphocytes, CD19+ B lymphocytes, and combined CD4+T lymphocytes and CD19+ B lymphocytes show accuracies similar to those of more complex scoring systems such as the Ranson score and APACHE II score.

AP is a common inflammatory disease. Respiratory, circulatory, and renal failure are the most important causes of AP death [19]. Although there are many scoring systems that can predict their prognosis, they have major drawbacks [7]. Currently, there is no single indicator that can predict OF. The occurrence of AP is often accompanied by alterations of the immune system. The activation of T and B lymphocytes is a key factor regulating the inflammatory response in different diseases, including AP [20]. When the inflammatory reaction in AP occurs, T lymphocytes are transformed into lymphoblasts and then differentiate into sensitized T lymphocytes, which play an anti-infective role in cellular immunity [21]. Similarly, B lymphocytes are first transformed into plasmablasts and then differentiate into plasma cells [8]. These cells participate in humoural immunity by producing and secreting immunoglobulins (antibodies) [22]. The role of different lymphocytes in AP was partly reported previously, but the mechanisms are still poorly understood. In a previous study, Curley et al. found that the proportion of CD4 + T lymphocytes in severe pancreatitis was significantly reduced and complications such as pseudocysts, local necrosis, and abscess formation occurred [23]. Yao Liu et al. noted that, in the early stage of SAP, the reduction in CD4+ T lymphocytes was closely associated with abdominal syndrome in AP [12], and it has also been found that that knockout of CD4+ T lymphocytes in mice significantly reduced the severity of their AP [24]. Therefore, a certain relationship between the activation of T lymphocytes and the progression of AP is believed to exist, although the function of peripheral blood CD4 + T lymphocytes and CD19+B lymphocytes in AP is still unclear.

To our knowledge, activation of circulating lymphocytes, both CD4+T lymphocytes and CD19+B lymphocytes, is a normal response to inflammation and is more likely to enhance the system's resistance to infection. However, excessive or uncontrolled activation may release toxic mediators, such as cytokines and oxygen free radicals [25]. CD4+ and CD8+ lymphocytes act as two major subsets of T lymphocytes, also known as T helper lymphocytes and cytotoxic T lymphocytes (CTLs). The number of CD4+ lymphocytes was significantly depleted in AP patients with OF, although the number of CD8+ lymphocytes was similar in both the NOF and OF groups. However, B lymphocytes were markedly increased in AP patients with OF compared with those in patients with NOF. These results suggest that T lymphocytes with the phenotype marker CD4+ are Th lymphocytes critical to the innate immune system and secrete anti-inflammatory cytokines, such as interleukin (IL)-10 and transforming growth factor (TGF)-*β* [26, 27]. We speculate that when AP is present, the proportion of CD4+T lymphocytes in the body decreases more significantly, which may suggest immunosuppression. The cause of this reduction in cell populations may be related to increased apoptosis of lymphocytes and homing of intestinal lymphocytes after pancreatitis occurs [28, 29]. Previously, in a mouse model of pancreatitis, pancreatic oedema, amylase, and pathological scores of B-cell-deficient mice were found to be significantly increased, indicating that B lymphocytes can inhibit inflammation and reduce pancreatic damage in AP. B lymphocytes are generally believed to have immunomodulatory functions, as well as inhibit the activation and proliferation of other inflammatory cells by secreting anti-inflammatory factors or antibodies [30, 31] and present antigens [22]. Interestingly, in this investigation, the CD19+B lymphocyte data may be of value as a reference for predicting the development of OF. The greater the numbers of activated CD19+B lymphocytes were, the more severe the inflammatory response was, and the more likely OF was to occur. When all AP cases were combined, CD4+T lymphocytes, CD19+B lymphocytes, and combined CD4+ and CD19+lymphocytes were of higher value in predicting AP OF. After considering the aetiology of AP, the predictive effect was also obvious. These predictors are easier to implement compared with complex scoring systems. Whether the immunological alterations observed in B lymphocytes are related to the pathogenesis of different causes of AP cannot be answered at present. Our findings suggest a fundamental difference in the pathophysiology and mechanism of biliary AP and hyperlipidaemia AP. Biliary pancreatitis is caused by obstruction of the pancreatic duct due to gallbladder or biliary stones, and then the secretion by the upper pancreas is blocked [32]. Hyperlipidaemia pancreatitis is caused by high TG levels and accumulation of oxidation products, calcium overload, etc [33, 34]. These factors may activate B lymphocytes and inhibit harmful inflammatory responses. However, the exact mechanism needs further clarification.

In summary, CD4+T lymphocytes and CD19+B lymphocytes are introduced as easily measurable parameters that can be used to assess OF in AP patients. However, our research has some limitations. In this study, we studied only Chinese people, who may have differences in lymphocytes compared with other populations, and the number of patients was limited (n = 133). Further, we did not compare the AP patients with healthy controls. Additionally, since there may be different changes in immune function during the occurrence and development of AP, separate tests may need to be performed at different stages of AP. This study investigated only immune function at admission and did not dynamically track changes in peripheral blood lymphocyte subsets during hospitalization. Although we analysed peripheral blood lymphocyte subsets across different causes of AP, the sample size was small. Studies with larger sample sizes should be further conducted to investigate the true value of CD4+ T and CD19+B lymphocytes in predicting AP OF. Therefore, further study is needed to confirm these observations.

## 5. Conclusion

Excessive or uncontrolled circulating lymphocyte activation may be important in the development of multiple OF. Patients with lower CD4+ lymphocyte counts and increased peripheral CD19+B lymphocyte levels at admission may have a higher risk of developing OF in AP, and these indicators appear to be novel predictors of OF in AP.

## Figures and Tables

**Table 1:** Peripheral blood lymphocyte subsets of AP Patients.

	**ALL**	**Normal Range**
No	133	
CD3+ T lymphocytes (%)	66.00 (56.69-73.19)	64-76
CD4+ T lymphocytes (%)	38.20 (31.19-45.31)	30-40
CD8+ cytotoxic T lymphocytes (%)	21.62 (17.27-26.39)	20-30
CD16+CD56+ natural killer cells (%)	12.07 (8.57-17.78)	10-20
CD19+ B lymphocytes (%)	16.80 (11.35-23.01)	9-14
CD4+/CD8+	1.79 (1.25-2.43)	1-2.5

Dates are presented as the median (25th-75th percentile).

**Table 2:** Basic characteristic of AP patients.

	**ALL**	**NOF**	**OF**	**P-value**
No	133	109	24	
Age, years	56.62 ± 17.17	50.56 ± 15.45	44.33 ± 18.56	0.087
Gender, M/F	79/54	66/43	13/11	0.568
Current smoker	20 (15.1%)	19 (17.4%)	1 (4.2%)	0.2
Hypertension	39 (29.3%)	31 (28.4%)	8 (33.3%)	0.637
Diabetes mellitus	19 (9.8%)	15 (13.8%)	4 (16.7%)	0.728
Etiology				0.971
Biliary	91 (68.4%)	75 (68.8%)	16 (66.7%)	
Alcoholic	13 (9.8%)	11 (10.1%)	2 (8.3%)	
Hyperlipidemia	23 (17.3%)	18 (16.5%)	5 (20.8%)	
Idiopathic	6 (4.5%)	5 (4.6%)	1 (4.2%)	

Dates are presented in either means and standard deviations or frequencies and percentages. Student's t test and chi-square test are used.

**Table 3:** Peripheral blood lymphocytes subsets in total patients with AP.

	**NOF (**%**)**	**OF (**%**)**	**P-value**
CD3+ T lymphocytes (%)	66.50 (57.45-73.70)	61.31 (51.18-72.38)	0.133
CD4+ T lymphocytes (%)	39.60 (33.94-46.13)	32.41 (26.51-38.00)	0.004
CD8+ Cytotoxic T lymphocytes (%)	21.62 (17.27-26.18)	21.63 (17.33-26.94)	0.847
CD16+CD56+ Natural killer cells (%)	12.14 (8.93-17.96)	11.07 (6.09-16.48)	0.343
CD19+ B lymphocytes (%)	16.07 (10.67-21.06)	23.78 (17.84-29.45)	0.001
CD4+/CD8+	1.82 (1.30-2.51)	1.61 (1.11-2.10)	0.180

Dates are presented in median (25th-75th percentile). Mann-Whitney U test is used.

**Table 4:** Peripheral blood lymphocytes subsets in biliary pancreatitis.

	**NOF (**%**)**	**OF (**%**)**	**P-value**
CD3+ T lymphocytes (%)	66.50 (56.82-72.40)	61.31 (48.30-72.38)	0.219
CD4+ T lymphocytes (%)	38.44 (32.51-46.62)	32.41 (28.50-38.84)	0.040
CD8+ Cytotoxic T lymphocytes (%)	21.12 (16.96-25.43)	20.76 (15.22-25.98)	0.855
CD16+CD56+ Natural killer cells (%)	12.14 (8.79-17.84)	11.07 (8.31-21.66)	0.770
CD19+ B lymphocytes (%)	16.07 (11.00-21.53)	24.37 (17.84-28.95)	0.015
CD4+/CD8+	1.91 (1.28-2.62)	1.84 (1.19-2.47)	0.628

Dates are presented in median (25th-75th percentile). Mann-Whitney U test is used.

**Table 5:** Peripheral blood lymphocytes subsets in alcoholic pancreatitis.

	**NOF (**%**)**	**OF (**%**)**	**P-value**
CD3+ T lymphocytes (%)	72.50 (61.44-76.69)	53.50 (/)	0.076
CD4+ T lymphocytes (%)	44.12 (38.31-51.81)	27.96 (/)	0.048
CD8+ Cytotoxic T lymphocytes (%)	22.06 (17.12-28.50)	24.06 (/)	0.693
CD16+CD56+ Natural killer cells (%)	14.40 (9.29-17.07)	14.65 (/)	0.693
CD19+ B lymphocytes (%)	11.64 (8.86-18.53)	26.76 (/)	0.076
CD4+/CD8+	2.34 (1.41-2.58)	1.15 (/)	0.076

Dates are presented in median (25th-75th percentile). Mann-Whitney U test is used.

**Table 6:** Peripheral blood lymphocytes subsets in hyperlipidemia pancreatitis.

	**NOF (**%**)**	**OF (**%**)**	**P-value**
CD3+ T lymphocytes (%)	66.38 (60.31-74.51)	63.31 (54.88-72.72)	0.412
CD4+ T lymphocytes (%)	39.92 (29.94-44.82)	27.88 (26.00-37.19)	0.037
CD8+ Cytotoxic T lymphocytes (%)	22.89 (19.17-27.76)	24.31 (18.41-27.28)	0.881
CD16+CD56+ Natural killer cells (%)	11.34 (8.89-23.36)	7.64 (5.25-17.30)	0.264
CD19+ B lymphocytes (%)	16.39 (11.15-19.70)	21.40 (16.43-30.29)	0.053
CD4+/CD8+	1.57 (1.25-2.08)	1.39 (1.05-1.69)	0.280

Dates are presented in median (25th-75th percentile). Mann-Whitney U test is used.

**Table 7:** ROC analysis in diagnosing OF.

	APACHE-II	Ranson	CD19+B lymphocytes	CD4+T lymphocytes	combined CD4+ and CD19+
Total pancreatitis	0.78	0.72	0.72	0.69	0.73
	(0.69-0.88)	(0.62-0.82)	(0.61-0.84)	(0.57-0.81)	(0.61-0.86)
Biliary pancreatitis	0.83	0.80	0.70	0.66	0.71
	(0.73-0.93)	(0.70-0.90)	(0.55-0.84)	(0.52-0.81)	(0.56-0.85)
Alcoholic pancreatitis	0.66	0.64	0.91	0.96	0.91
	(0.29-1.00)	(0.34-1.00)	(0.74-1.00)	(0.83-1.00)	(0.72-1.00)
Hyperlipidemia pancreatitis	0.60	0.54	0.79	0.81	0.83
	(0.31-1.00)	(0.26-0.82)	(0.55-1.00)	(0.64-0.99)	(0.58-1.00)

95% Cl included. AUC: area under the curve; Cl: confidence intervals.

## Data Availability

The data used to support the findings of this study are available from the authors upon request.

## References

[1] Wang G.-J., Gao C.-F., Wei D., Wang C., Ding S.-Q. (2009). Acute pancreatitis: Etiology and common pathogenesis. *World Journal of Gastroenterology*.

[2] Banks P. A., Bollen T. L., Dervenis C. (2013). Classification of acute pancreatitis--2012: revision of the Atlanta classification and definitions by international consensus. *Journal of Clinical Hepatology*.

[3] Renner I. G., Savage W. T., Pantoja J. L., Renner V. J. (1985). Death due to acute pancreatitis. A retrospective analysis of 405 autopsy cases. *Digestive Diseases and Sciences*.

[4] Pavlidis T. E., Pavlidis E. T., Sakantamis A. K. (2010). Advances in prognostic factors in acute pancreatitis: A mini-review. *Hepatobiliary & Pancreatic Diseases International*.

[5] McMahon M. J., Playforth M. J., Pickford I. R. (1980). A comparative study of methods for the prediction of severity of attacks of acute pancreatitis. *British Journal of Surgery*.

[6] Knaus W. A., Draper E. A., Wagner D. P., Zimmerman J. E. (1985). APACHE II: a severity of disease classification system. *Critical Care Medicine*.

[7] Sarr M. G. (2013). 2012 revision of the Atlanta classifcation of acute pancreatitis. *Polskie Archiwum Medycyny Wewnętrznej*.

[8] Denman A. M. (2015). *Cellular and Molecular Immunology*.

[9] Zheng L., Xue J., Jaffee E. M., Habtezion A. (2013). Role of immune cells and immune-based therapies in pancreatitis and pancreatic ductal adenocarcinoma. *Gastroenterology*.

[10] Marja-Leena K., Heikki P., Antero P. (2010). Inflammation and immunosuppression in severe acute pancreatitis. *World Journal of Gastroenterology*.

[11] Sun J., Bhatia M. (2007). Blockade of neurokinin-1 receptor attenuates CC and CXC chemokine production in experimental acute pancreatitis and associated lung injury. *American Journal of Physiology-Gastrointestinal and Liver Physiology*.

[12] Liu Y., Wang L., Cai Z. (2015). The decrease of peripheral blood CD4+ T cells indicates abdominal compartment syndrome in severe acute pancreatitis. *PLoS ONE*.

[13] Banks P. A., Bollen T. L., Dervenis C. (2013). Classification of acute pancreatitis—2012: revision of the Atlanta classification and definitions by international consensus. *Journal of Clinical Hepatology*.

[14] Uk W. (2010). UK guidelines for the management of acute pancreatitis. *Gut*.

[15] Group of Pancreas Surgery CSoS (2007). The guideline of diagnosis and treatment of severe acute pancreatitis. *Chinese Journal of Surgery*.

[16] Klinker M. W., Lundy S. K. (2012). Multiple mechanisms of immune suppression by B lymphocytes. *Molecular Medicine*.

[17] Zhang Y., Luo F., Cai Y. (2011). TLR1/TLR2 agonist induces tumor regression by reciprocal modulation of effector and regulatory T cells. *The Journal of Immunology*.

[18] Beger H. G., Gansauge F., Mayer J. M. (2000). The role of immunocytes in acute and chronic pancreatitis: When friends turn into enemies. *Gastroenterology*.

[19] Frossard J. L., Steer M. L., Pastor C. M. (2008). Acute pancreatitis. *The Lancet*.

[20] Mora A., Pérez-Mateo M., Viedma J. A., Carballo F., Sánchez-Payá J., Liras G. (1997). Activation of cellular immune response in acute pancreatitis. *Gut*.

[21] Klabusay M. (2015). The role of regulatory T-cells in antitumor immune response. *Journal of the Czech and Slovak Oncological Societies*.

[22] Mauri C., Bosma A. (2012). Immune regulatory function of B cells. *Annual Review of Immunology*.

[23] Curley P. J., McMahon M. J., Lancaster F. (1993). Reduction in circulating levels of CD4‐positive lymphocytes in acute pancreatitis: Relationship to endotoxin, interleukin 6 and disease severity. *British Journal of Surgery*.

[24] Demols A., Le Moine O., Desalle F., Quertinmont E., Van Laethem J.-L., Devière J. (2000). CD4+ T cells play an important role in acute experimental pancreatitis in mice. *Gastroenterology*.

[25] Kleeff J., Friess H. (1997). Immune function early in acute pancreatitis. *Zeitschrift für Gastroenterologie*.

[26] Chinen T., Volchkov P. Y., Chervonsky A. V., Rudensky A. Y. (2010). A critical role for regulatory T cell-mediated control of inflammation in the absence of commensal microbiota. *The Journal of Experimental Medicine*.

[27] Jia W., Cao L., Yang S. (2013). Regulatory T cells are protective in systemic inflammation response syndrome induced by zymosan in mice. *PLoS ONE*.

[28] Wittel U. A., Rau B., Gansauge F. (2004). Influence of PMN leukocyte-mediated pancreatic damage on the systemic immune response in severe acute pancreatitis in rats. *Digestive Diseases and Sciences*.

[29] Pietruczuk M., Dabrowska M. I., Wereszczynska-Siemiatkowska U., Dabrowski A. (2006). Alteration of peripheral blood lymphocyte subsets in acute pancreatitis. *World Journal of Gastroenterology*.

[30] Gray D., Gray M. (2010). What are regulatory B cells?. *European Journal of Immunology*.

[31] Mauri C. (2010). Regulation of immunity and autoimmunity by B cells. *Current Opinion in Immunology*.

[32] Lankisch P. G., Apte M., Banks P. A. (2015). Acute pancreatitis. *The Lancet*.

[33] Pereda J., Pérez S., Escobar J., Arduini A., Asensi M., Serviddio G. (2012). Obese Rats Exhibit High Levels of Fat Necrosis and Isoprostanes in Taurocholate-Induced Acute Pancreatitis. *PLoS ONE*.

[34] Yang F., Wang Y., Sternfeld L. (2009). The role of free fatty acids, pancreatic lipase and Ca2+ signalling in injury of isolated acinar cells and pancreatitis model in lipoprotein lipase-deficient mice. *Acta Physiologica*.

